# Prospective international validation of the predisposition, infection, response and organ dysfunction (PIRO) clinical staging system among intensive care and general ward patients

**DOI:** 10.1186/s13613-021-00966-7

**Published:** 2021-12-23

**Authors:** T. Cardoso, P. P. Rodrigues, C. Nunes, M. Almeida, J. Cancela, F. Rosa, N. Rocha-Pereira, I. Ferreira, F. Seabra-Pereira, P. Vaz, L. Carneiro, C. Andrade, J. Davis, A. Marçal, N. D. Friedman

**Affiliations:** 1 Intensive Care Unit (UCIP) and Hospital Infection Control Committee, Hospital de Santo António, Oporto University Hospital Center, University of Porto, Largo Prof. Abel Salazar, 4099-001 Porto, Portugal; 2 Department of Community Medicine, Information and Health Decision Sciences & CINTESIS, Faculty of Medicine, University of Porto, Rua Dr. Plácido Costa, s/n, 4200-450 Porto, Portugal; 3 Intensive Care Unit and Hospital Infection Control Committee, Hospital de Bragança, Northeastern Local Health Unit, Av. Abade Baçal, 5301-852 Bragança, Portugal; 4 Neurocritical Care Unit and Hospital Infection Control Committee, Hospital de São Marcos, Sete Fontes – São Vitor, 4710-243 Braga, Portugal; 5 Internal Medicine Department, Hospital Pedro Hispano, Matosinhos Local Health Unit, R. Dr. Eduardo Torres, Sra. da Hora, Portugal; 6 Infectious Diseases Department, São João Hospital Center, Alameda Prof. Hernâni Monteiro, 4200-319 Porto, Portugal; 7 Internal Medicine Department, Hospital de Santo António, Oporto University Hospital Center, Largo Prof. Abel Salazar, 4099-001 Porto, Portugal; 8 Intensive Care Unit (UCIP), Hospital de Santo António, Oporto University Hospital Center, Largo Prof. Abel Salazar, 4099-001 Porto, Portugal; 9 Internal Medicine Department and Hospital Infection Control Committee, Hospital de Bragança, Northeastern Local Health Unit, Av. Abade Baçal, 5301-852 Bragança, Portugal; 10 Department of Renal Medicine, Barwon Health, Geelong, VIC 3220 Australia; 11 Internal Medicine Department, Hospital Pedro Hispano, Matosinhos Local Health Unit, R. Dr. Eduardo Torres, Sra. da Hora, Portugal; 12 Department of Infectious Diseases, Barwon Health, Geelong, VIC 3220 Australia; 13 Present Address: Intensive Care Unit (UCIP), Hospital de Santo António, Oporto University Hospital Center, Largo Prof. Abel Salazar, 4099-001 Porto, Portugal; 14 Present Address: Intensive Care Unit and Internal Medicine Department, Hospital da Prelada, Rua de Sarmento de Beires, 4250-449 Porto, Portugal; 15 Present Address: Internal Medicine Department, Hospital de Santo António, Oporto University Hospital Center, Largo Prof. Abel Salazar, 4099-001 Porto, Portugal

**Keywords:** Predisposition, Infection, Response, Organ dysfunction, PIRO staging system, Hospital mortality

## Abstract

**Background:**

Stratifying patients with sepsis was the basis of the predisposition, infection, response and organ dysfunction (PIRO) concept, an attempt to resolve the heterogeneity in treatment response. The purpose of this study is to perform an independent validation of the PIRO staging system in an international cohort and explore its utility in the identification of patients in whom time to antibiotic treatment is particularly important.

**Methods:**

Prospective international cohort study, conducted over a 6-month period in five Portuguese hospitals and one Australian institution. All consecutive adult patients admitted to selected wards or the intensive care, with infections that met the CDC criteria for lower respiratory tract, urinary, intra-abdominal and bloodstream infections were included.

**Results:**

There were 1638 patients included in the study. Patients who died in hospital presented with a higher PIRO score (10 ± 3 vs 8 ± 4, *p* < 0.001). The observed mortality was 3%, 15%, 24% and 34% in stage I, II, III and IV, respectively, which was within the predicted intervals of the original model, except for stage IV patients that presented a lower mortality. The hospital survival rate was 84%. The application of the PIRO staging system to the validation cohort resulted in a positive predictive value of 97% for stage I, 91% for stage II, 85% for stage III and 66% for stage IV. The area under the receiver operating characteristics curve (AUROC) was 0.75 for the all cohort and 0.70 if only patients with bacteremia were considered. Patients in stage III and IV who did not have antibiotic therapy administered within the desired time frame had higher mortality rate than those who have timely administration of antibiotic.

**Conclusions:**

To our knowledge, this is the first external validation of this PIRO staging system and it performed well on different patient wards within the hospital and in different types of hospitals. Future studies could apply the PIRO system to decision-making about specific therapeutic interventions and enrollment in clinical trials based on disease stage.

**Supplementary Information:**

The online version contains supplementary material available at 10.1186/s13613-021-00966-7.

## Background

Infections are one of the five leading causes of death worldwide [1], representing a major contribution to the overall health care burden [2]. The worldwide “Surviving Sepsis Campaign” (SSC), achieved a reduction in mortality from severe sepsis and septic shock of 25% in 5 years by setting recommendations grouped into “bundles” based on time of performance. It included; early recognition, early use of antibiotics, early goal-directed therapy resuscitation protocol, and early supportive care in the ICU [3]. Nonetheless, the incidence of sepsis continues to increase as do the number of absolute deaths, despite improved survival rates [4, 5].

The development of new adjunctive therapies for sepsis, including molecular therapeutic or immunomodulatory agents, has unfortunately resulted in failure to demonstrate effectiveness which have been attributed to the inclusion of a very heterogeneous group of patients [6].

Stratifying patients with sepsis into a more homogeneous population that could benefit from specific therapies was the basis of the Predisposition, Infection, Response and Organ dysfunction (PIRO) concept [7]. This concept describes the phenotype of a patient with sepsis based on those four dimensions [8].

Several studies have been developed to test this concept in multiple settings with the ultimate goal of developing a score [9–13] or a model [14–16] that would predict mortality from sepsis. In 2013 we published a clinical staging system based on the PIRO concept [6], including in Predisposition age, several comorbidities, functional status and previous ATB therapy; in Infection, type of infection (either community, healthcare or hospital-acquired); in Response altered temperature, hyperglycemia, tachypnea and severity of infection and in Organ dysfunction, hypotension and SOFA score on the moment of diagnosis. The original study showed good discrimination in predicting hospital mortality, and allowed classification of patients according to their PIRO phenotype. That was a single center study. The purpose of the current study is to preform external validation of the PIRO staging system in an international prospective cohort of hospitalized patients with infection.

## Methods

### Study design and population

Prospective international cohort study, conducted over a 6-month period (1st October 2014 to 31st March 2015) in five Portuguese hospitals (three teaching and tertiary care and two secondary care hospitals) and one Australian institution (teaching and tertiary care hospital). Data were collected in individual centers and entered directly into a web-based electronic case report form.

All consecutive adult patients admitted to selected wards or the intensive care unit (ward selection was by convenience) of participating hospitals with selected infections were included (Additional file 1: Table S1). Infections included those that met the Centers for Disease Control and Prevention’s (CDC) criteria for lower respiratory tract, urinary, intra-abdominal and bloodstream infection [17]. Primary bloodstream infections included intravascular device-associated infections.

#### Definitions

Secondary bloodstream infection was defined as an infection when an organism isolated from a blood culture was related to an infection at another site.

Community-acquired infection (CAI) was defined as an infection detected within 48 h of hospital admission in patients who do not fit the criteria for a healthcare-associated infection (HCAI).

HCAI was defined a priori using the criteria of Friedman and colleagues published in 2002 [18].

Hospital-acquired infection (HAI) was defined as a localized or systemic infection that occurred 48 h or more after hospital admission and was not incubating at the time of hospital admission [19]. Infections arising in patients discharged from the hospital within the previous 2-week period were also included in this group.

Previous antibiotic therapy was defined as any antibiotic administered in the previous month with therapeutic intent.

Time to antibiotic therapy was calculated between infection diagnosis time and antibiotic administration time, and then categorized into antibiotic administration within the first 1, 3 and 6 h.

Severity of infection (infection, sepsis, severe sepsis or septic shock) was defined according to the criteria proposed by the American College of Chest Physicians/Society of Critical Care Medicine [20].

### Staging system

The PIRO staging system is based on Predisposition, Infection, Response and Organ dysfunction scores. The original scores were developed according to the original proposal of the American Thoracic Society/Society of Critical care Medicine [7] and are shown in Table 1 [6]. Predisposition score [range, 0 (best) to 18 (worst) points] allows stratification into P_1_ (score 0–2), P_2_ (score 3–4) and P_3_ (score ≥ 5). The Infection score [range, 0 (best) to 2 (worst) points] allows stratification into I_1_ (score 0–1) and I_2_ (score 2). The Response score [range, − 1 (best) to 7 (worst) points] allows stratification into R_1_ (score – 1 to 3) and R_2_ (score ≥ 4). The Organ dysfunction score [range, 0 (best) to 4 (worst) points] allows stratification into O_1_ (score 0) and O_2_ (score ≥ 1).

**Table 1:** Scores attributed to the selected variables included in each of the four components of PIRO [6]

P score	Points	I score	Points	R score	Points	O score	Points
*Age, years*		*Type of infection*		*Altered temperature*		Hypotension	3
≤ 60	0	CAI	0	No	0	SOFA > 0	1
61–80	1	HCAI	1	Fever	− 1		
> 80	3	HAI	2	Hypothermia	1		
Male	1			Hyperglycemia	1		
Previous ATB	1			Tachypnea	1		
Chronic hepatic disease	4			*Severity of infection*			
Chronic hematologic disease	3			Infection or sepsis	0		
Cancer	3			Severe sepsis	1		
Atherosclerosis	1			Septic shock	4		
Karnovsky index < 70	2						
Total possible points	18		2		7		4
P1 (0–2 point)		I1 (0–1 point)		R1 (-1–3 points)		O1 (0 points)	
P2 (3–4 points)		I2 (2 points)		R2 (≥ 4 points)		O2 (≥ 1points)	
P3 (≥ 5 points)							

*P-score* predisposition score, *I score* insult/infection score, *R score* host response score, *O score* organ dysfunction score, *ATB* antibiotic therapy, *CAI* community-acquired infection, *HCAI* healthcare-associated infection, *HAI* hospital-acquired infection, *SOFA* sepsis-related organ failure assessment

The PIRO score was computed as soon as the patient was enrolled in the study, up to 24 h maximum after the clinical diagnosis of infection. Any missing value was attributed the value of 0 for the final calculus.

The phenotypes that compose each stage and the predicted mortality are shown in Table 2 [6].The original cohort published in 2013 did not contemplate all possible phenotypes, namely: P1I2R2O1 and P1I1R2O1 that present an hospital mortality rate of 0% and were included in stage I; P2I2R2O1, with a hospital mortality rate of 13% and P1I2R2O2 and P2I1R2O1, with an hospital mortality rate of 14%, that were included in stage II; and P3I1R2O1, with a mortality rate 33% and P3I2R2O1 with 50%, that were included in stage III.

**Table 2:** Distribution of patients by each stage according to their phenotype, predicted hospital mortality in the original model [6] and the observed mortality in the validation cohort (*n* = 1638)

Focus of infection	Stage I (*n* = 431, 26%)	Stage II (*n* = 510, 31%)	Stage III (*n* = 601, 37%)	Stage IV (*n* = 96, 6%)
	Predicted hospital mortality rate 0–5%	Predicted hospital mortality rate 6–20%	Predicted hospital mortality rate 21–50%	Predicted hospital mortality rate 51–100%
	P_1-2_ I_1-2_ R_1_ O_1_	P_1_ I_2_ R_1_ O_2_		P_2-3_ I_1-2_ R_2_ O_2_
	P_1_ I_1_ R_1_ O_2_	P_1_ I_1-2_ R_2_ O_2_	P_3_ I_1-2_ R_2_ O_1_	
	P_1_ I_1-2_ R_2_ O_1_	P_2_ I_1-2_ R_1_ O_2_	P_3_ I_1-2_ R_1_ O_2_	
	P_2_ I_1-2_ R_1_ O_1_	P_2_ I_1-2_ R_2_ O_1_		
		P_3_ I_1-2_ R_1_ O_1_		

	Observed hospital mortality rate 3% (*n* = 14)	Observed hospital mortality rate 15% (*n* = 78)	Observed hospital mortality rate 24% (*n* = 145)	Observed hospital mortality rate 34% (*n* = 33)
Respiratory (*n* = 860, 52.5%)	3% (6/215)	19% (46/248)	26% (94/363)	44% (15/34)
Urinary (*n* = 332, 20.3%)	3% (2/66)	10% (11/111)	15% (21/136)	26% (5/19)
GI (*n* = 282, 17.5%)	3% (4/126)	12% (11/91)	29% (12/41)	28% (8/29)
Primary bacteraemia (*n* = 159, 9.7%)	8% (2/24)	17% (10/60)	30% (18/61)	36% (5/14)

The performance of the current model to predict hospital mortality was compared against SAPS II, a physiological score designed to predict hospital mortality among intensive care patients [21], extrapolated to the various settings were patients were recruited.

### Statistical analysis

Categorical variables were described as proportions and compared using Chi-square or Fisher’s exact test. Continuous variables were described by mean and standard deviation. Comparisons of continuous variables were performed using Student’s *t-*test.

For the validation of each stage, the test was considered positive if the patient met the criteria for that stage. The outcome was survival at hospital discharge.

The area under the receiver operating characteristics curve was used to compare PIRO and SAPS II scores in different populations.

Statistical significance was defined as *p* < 0.05. The statistical analysis was performed in SPSS^®^24 (SPSS Inc., Chicago IL).

## Results

During the study period 1638 patients met the inclusion criteria. General characteristics of these patients are shown in Additional file 1: Table S2 and compared with the original cohort [6].

In Predisposition, the validation cohort included older patients, more males, more patients with cancer, atherosclerosis and a Karnofsky score < 70, less patients with hematologic disease and overall a higher P score. In Infection, the validation cohort included a lower prevalence of community and hospital-acquired infection and a greater prevalence of HCAI, with no difference in the overall I score. In Response, the validation cohort included a higher prevalence of fever, less hypothermia, more tachypnea and more severe sepsis, with no difference in the overall R score. In Organ dysfunction, the validation cohort included more hypotension and higher SOFA scores, with higher overall O score. The total PIRO score was higher in the validation cohort. Patients that died presented with a higher PIRO score in both cohorts (Additional file 1: Table S2).

In the current cohort patients who died in hospital presented with a higher PIRO score than those who survived (10 ± 3 vs 8 ± 4, *p* < 0.001).

In Table 2 the distribution of patients according to the four PIRO stages is shown along with the predicted and observed mortality. In the validation cohort, the observed mortality was within the predicted mortality of the original model, with the exception of stage IV, which had a lower mortality than predicted. The hospital mortality in each stage for different focus of infection was also within the predicted intervals, with exception of those in stage IV that presented a lower mortality than predicted regardless of the focus of infection; patients in stage I with primary bacteremia showed an hospital mortality of 8%, slightly higher than predicted (0–5%) and those in stage III with urinary infection lower than predicted (21–50%)–15%.

The prevalence of hospital survival in this population was 84%. The performance of the model for predicting hospital survival at each stage is illustrated in Table 3.

**Table 3:** Performance of the PIRO staging system for predicting hospital survival in each stage

	Stage I (*n* = 431)	Stage II (*n* = 510)	Stage III (*n* = 601)	Stage IV (*n* = 96)
Sensitivity, 95% CI	30% (28–33)	62% (59–65)	95% (83–86)	5% (4–6)
Specificity, 95% CI	95% (91–97)	66% (60–72)	12% (9–17)	88% (83–91)
Negative likelihood ratio, 95% CI	0.7 (0.7–0.8)	0.6 (0.5–0.6)	0.4 (0.3–0.6)	1.1 (1.0–1.1)
Positive likelihood ratio, 95% CI	5.9 (3.5–9.9)	1.8 (1.5–2.2)	1.1 (1.0–1.1)	0.4 (0.3–0.6)
Negative predictive value, 95% CI	21% (20–21)	25% (23–27)	34% (25–43)	18% (16–20)
Positive predictive value, 95% CI	97% (95–98)	91% (89–92)	85% (84–86)	66% (57–75)

*CI* confidence interval

The application of the PIRO staging system to the validation cohort resulted in a probability post-positive test (that is the probability of hospital survival after classification in each stage) of 97% for stage I, 91% for stage II, 85% for stage III and 66% for stage IV. Specificity was 95%, 66%, 12% and 88%, respectively, for stages I, II, III and IV (Table 3).

The area under the ROC curve (AUC) for hospital mortality was 0.75 for the PIRO model, and was similar if only patients with microbiological documentation of infection (0.74) or bacteremia (0.70) were considered. The AUC was higher for patients on the general ward (0.76) than in the ICU/HDU (0.68). The discrimination of the model was higher than SAPS II in all settings (Table 4).

**Table 4:** Area under the receiver operating characteristics curve (95% confidence interval) of the PIRO and SAPS II scores, in the original and in the validation cohorts

	PIRO	SAPS II
Validation cohort (*n* = 1638)	0.75 (0.72–0.78)	0.71 (0.68–0.75)
Microbiological documented (*n* = 1061)	0.74 (0.71–0.78)	0.72 (0.68–0.76)
Bacteremia (*n* = 302)	0.70 (0.63–0.78)	0.69 (0.62–0.77)
Ward (*n* = 1435)	0.76 (0.72–0.79)	0.71 (0.67–0.74)
ICU/HDU (*n* = 203)	0.68 (0.60–0.76)	0.67 (0.59–0.75)
Original cohort (*n* = 1035) [6]	0.85 (0.82–0.88)	0.81 (0.77–0.84)

*ICU* intensive care unit, *HDU* high-dependency unit

In Table 5 hospital mortality rate in each stage according to time of antibiotic therapy administration is shown; there were no significant differences in outcome, but there was a consistent trend towards lower hospital mortality rate in patients with earlier administration of antibiotic therapy particularly in those in stage III and IV. There were seven patients in whom time of antibiotic administration was not registered and therefore not included in this analysis.

**Table 5:** Mortality rate by stage according to time from diagnosis to antibiotic therapy

	ATB within the first hour		ATB within the first 3 h		ATB within the first 6 h	
	Yes	No	Yes	No	Yes	No
Stage I *n* = 427	4.1% (10/241)	2.2% (4/186)	3.6% (11/307)	2.5% (3/120)	3.4% (12/357)	2.9% (2/70)
*p* value	0.286^#^		0.572^#^		1.000^#^	
Stage II *n* = 508	16.1% (45/279)	14.4% (33/229)	15.6% (56/359)	14.8% (22/149)	15.6% (66/422)	14.0% (12/86)
*p* value	0.593*		0.812*		0.693*	
Stage III *n* = 600	23.8% (74/311)	24.2% (70/289)	22.5% (96/426)	27.6% (48/174)	23.1% (116/503)	28.9% (28/97)
*p* value	0.903*		0.189*		0.220*	
Stage IV *n* = 96	25% (10/40)	41.1% (23/56)	23.1% (6/26)	38.6% (27/70)	16.7% (3/18)	38.5% (30/78)
*p* value	0.102^*^		0.155^*^		0.102^#^	

(number of deceased/total number of patients within the group)

*ATB* antibiotic therapy

^*^Chi-square test

^#^Fisher’s exact test

## Discussion

In this study, we present the first external validation of the PIRO staging system [6]. In this international study, the observed hospital mortality rate in each stage was within the range predicted by our original work, with increasing mortality from stage I to stage III. Patients in stage IV exhibited the highest mortality rate, albeit lower than predicted, which may reflect the overall decrease in mortality attributed to sepsis and severe sepsis [4, 5]. The performance of this staging model was similar for all studied focus of infection, reinforcing its applicability in different infections.

There were some differences between the original cohort and the current one, namely in different components of predisposition, insult, response and organ dysfunction that would alter the distribution of patients in each phenotype and stage. Nevertheless, although the proportion of patients in each stage was different, the outcome remained within the previsions of the original work in each stage, except for stage IV that presented a lower mortality in the current study, suggesting that it would perform equally well in different populations.

As in the original cohort, we found that patients who died in hospital displayed higher PIRO scores when compared with those that survived. The performance of the original model in the validation cohort (AUC 0.75) was moderately good and similar to other validation studies of different PIRO models [9, 10, 22–24].

Although there have been several PIRO models and scores previously published, our work is the first to validate a true staging system based on the PIRO concept. Among previously developed scoring systems based on the PIRO concept the most studied are from Howell et al. [8], Rubulotta et al. [16] and Moreno et al. [15].

Howell et al. prospectively studied Emergency Department (ED) patients and their model performed very well in the original cohort with an AUC of 0.90, while subsequent studies showed an AUC of between 0.73 [25] and 0.86 [26] in ED patients and 0.71 [22] to 0.75 [23], in ICU patients.

Rubulotta et al. [16] performed a secondary analysis of a large database of patients with severe sepsis (PROWESS and PROGRESS). Subsequent studies on ICU patients showed an AUC of 0.71 [24] and 0.76 [27], for ED patients and 0.65 [23] and 0.71 [22], for ICU patients.

Moreno et al. [15] also performed a secondary analysis of a database of ICU patients (SAPS 3) with an AUC of 0.77 in the original cohort, while subsequent studies showed an AUC of 0.74 [23] and 0.84 [22], both in ICU patients.

Our model also showed that PIRO performed better than SAPS II in ICU and in the general ward patients, increasing its range of applicability. Importantly, all required variables for the model can be collected almost immediately at the bedside which is imperative for early treatment and possible enrollment in clinical trials.

This staging system is a way to stratify patients into a more homogeneous groups and test the value of interventions that would reduce disease progression, morbidity and mortality. The ultimate aim of the PIRO staging system is to identify the appropriate patient population more likely to benefit from new and/or specific therapeutic interventions [28].

Although this study was not designed specifically to evaluate the impact of different therapies in each stage, we have tried to see if patients in different stages respond differently to timely administration of antibiotic therapy and, although not reaching statistical significance, the mortality rates in patients from stages III and IV were consistently higher in the group with delayed antibiotic administration, regardless of the time interval considered (1, 3 or 6 h), suggesting that these phenotypes are particularly sensitive to this therapeutic intervention. This is the final objective of this staging system: to elect patients who benefit the most from specific therapies, may that be early antibiotic therapy or new immunomodulatory agents.

Our study has various limitations that need to be acknowledged. As a prospective observational study, ascertainment and informational bias cannot be excluded. Our study includes only one biomarker (C-reactive protein) although there are other biomarkers already in use, like procalcitonin that could assist with discrimination. Furthermore, 35% of patients did not have microbiological documentation of infection, however, the analysis of the subgroup with positive blood cultures revealed a similar performance. The small number of patients included in stage IV might explain the low sensibility of this group, a larger study or one focused on patients with severe infection might help to improve the sensibility of the staging system in this group.

Nonetheless, the strengths of this study are the fact that it is an international prospective cohort study, with a large cohort size, incorporating both community and university hospitals, and it includes patients from different wards in the hospital which increases its external applicability.

Patients with very similar mortality risk may have dramatically different responses to therapy based on their PIRO stage. It is hoped that this study makes a contribution towards translation of the PIRO concept into clinical practice.

## Conclusions

To our knowledge, our study is the first external validation of the PIRO staging system and it showed good performance in different settings within the hospital and in different types of hospitals. Future studies could apply the PIRO system to decision-making with respect to primary and adjuvant treatment modalities based on disease stage at clinical presentation.

## Supplementary Information

**Additional file 1: Table S1. **Characterization of participating hospitals and patients’ distribution by type of ward in each hospital. **Table S2. **Comparison of variables significantly associated with hospital mortality, within each of the four components of PIRO in the original and the validation cohorts.

## Data Availability

The datasets used and/or analyzed during the current study are available from the corresponding author on reasonable request.

## Abbreviations

AUROC: Area under the receiver operating characteristics curve
CAI: Community-acquired infection
CDC: Centers for Disease Control and Prevention
ED: Emergency department
HAI: Hospital-acquired infection
HCAI: Healthcare-associated infection
HDU: High-dependency unit
ICU: Intensive care unit
PIRO: Predisposition, infection, response and organ dysfunction
SAPS II: Simplified Acute Physiological Score
SOFA: Sequential Organ Failure Assessment
SSC: Surviving Sepsis Campaign
