# Supplementary material for: Prospective international validation of the predisposition, infection, response and organ dysfunction (PIRO) clinical staging system among intensive care and general ward patients
Source: Ann Intensive Care. 2021 Dec 23;11:180. doi: 10.1186/s13613-021-00966-7 (PMC8702585; doi:10.1186/s13613-021-00966-7)
Supplement: Supplementary file 1 — Additional file 1: Table S1. Characterization of participating hospitals and patients’ distribution by type of ward in each hospital. Table S2. Comparison of variables significantly associated with hospital mortality, within each of the four components of PIRO in the original and the validation cohorts. [file 13613_2021_966_MOESM1_ESM.docx]

**Additional file 1**

**Table S1. Characterization of participating hospitals and patients’ distribution by type of ward in each hospital.**

| **Hospital**  **(total number of patients included)** | **Number of patients admitted to Medical wards** | **Number of patients admitted to Surgical wards** | **Number of patients admitted to HDU/ICU** |
| --- | --- | --- | --- |
| 1 (n = 109) | 83 | 9 | 17 |
| 2 (n = 607) | 347 | 180 | 80 |
| 3 (n = 93) | 63 | 0 | 30 |
| 4 (n = 303) | 233 | 32 | 38 |
| 5 (n = 194) | 180 | 0 | 14 |
| 6 (n = 332) | 262 | 46 | 24 |
| **Total (n = 1638)** | **1168** | **267** | **203** |

1, 2, 3 and 4 – teaching and tertiary care hospital; 5 and 6 - secondary care hospitals

HDU – High dependency unit; ICU – Intensive care unit

**Table S2. Comparison of variables significantly associated with hospital mortality, within each of the four components of PIRO in the original and the validation cohorts.**

|  | Original cohort,  n= 1035 | | Validation cohort,  n= 1638 | | p value | |
| --- | --- | --- | --- | --- | --- | --- |
| Variables | Total, n(%) | Non-survivors, n (%) | Total, n(%) | Non-survivors, n (%) | Total | Non-survivors |
| **Predisposition** |  |  |  |  |  |  |
| Age, mean (SD) | 65±20 | 76±14 | 71±16 | 77±12 | <0.001 | 0.453 |
| Male sex, n (%) | 506 (49) | 79 (16) | 924 (56) | 167 (18) | 0.004 | 0.339 |
| Previous antibiotic therapy, n (%) | 367 (36) | 67 (18) | 619 (38) | 124 (20) | 0.298 | 0.441 |
| Chronic hepatic disease, n (%) | 22 (2) | 8 (36) | 43 (3) | 14 (33) | 0.115 | 0.635 |
| Chronic hematologic disease, n (%) | 60 (6) | 17 (28) | 59 (4) | 19 (32) | 0.018 | 0.225 |
| Cancer, n (%) | 45 (4) | 18 (40) | 97 (6) | 28 (29) | 0.024 | 0.194 |
| Atherosclerosis, n (%) | 242 (23) | 54 (22) | 558 (34) | 115 (21) | <0.001 | 0.816 |
| Karnovsky index<70, n (%) | 319 (31) | 81 (25) | 756 (46) | 171 (23) | <0.001 | 0.846 |
| Total P score, median (IQR) | 3 ± 2 | 5 ± 2 | 4 ± 2 | 5 ± 2 | <0.001 | 1.000 |
| **Infection** |  |  |  |  |  |  |
| Community-acquired, n (%) | 493 (48) | 47 (10) | 692 (42) | 69 (10) | 0.024 | 1.000 |
| Healthcare-associated, n (%) | 225 (22) | 32 (14) | 562 (34) | 98 (17) | <0.001 | 0.302 |
| Hospital-acquired, n (%) | 316 (30) | 59 (19) | 384 (24) | 103 (27) | 0.006 | 0.075 |
| Total I score, median (IQR) | 1 ± 1 | 1 ± 1 | 1 ± 1 | 1 ± 1 | 1.000 | 1.000 |
| **Response** |  |  |  |  |  |  |
| *Temperature* |  |  |  |  |  |  |
| No alteration, n(%) | 461 (45) | 57 (12) | 706 (43) | 121 (17) | 0.310 | 0.020 |
| Fever, n (%) | 336 (33) | 35 (10) | 764 (47) | 108 (14) | <0.001 | 0.002 |
| Hypothermia, n (%) | 238 (22) | 46 (19) | 168 (10) | 41 (24) | <0.001 | 0.269 |
| Tachypnea, n (%) | 457 (44) | 83 (18) | 782 (48) | 169 (22) | 0.043 | 0.346 |
| Hyperglycemia, n (%) | 159 (15) | 38 (24) | 226 (14) | 51 (23) | 0.473 | 0.822 |
| *Severity of infection* |  |  |  |  |  |  |
| Infection or sepsis, n (%) | 645 (62) | 50 (8) | 359 (22) | 21 (6) | <0.001 | 0.243 |
| Severe sepsis, n (%) | 296 (29) | 46 (15) | 1164 (71) | 208 (18) | <0.001 | 0.224 |
| Septic shock, n (%) | 94 (9) | 42 (45) | 115 (7) | 41 (36) | 0.060 | 0.188 |
| Total R score, median (IQR) | 1 ± 1 | 3 ± 2 | 1 ± 1 | 2 ± 1 | 1.000 | <0.001 |
| **Organ dysfunction** |  |  |  |  |  |  |
| Hypotension, n (%) | 175 (17) | 63 (36) | 385 (24) | 103 (27) | <0.001 | 0.061 |
| SOFA>0 | 691 (67) | 118 (17) | 1450 (89) | 256 (18) | <0.001 | 0.571 |
| Total O score, median (IQR) | 1 ± 1 | 2 ± 2 | 2 ± 1 | 2 ± 2 | <0.001 | 1.000 |
| **Total PIRO score** | 6 ± 4 | 11 ± 4 | 8 ± 4 | 10 ± 3 | <0.001 | <0.001 |

SD – standard deviation; IQR – inter-quartil range; SOFA – Sepsis-related Organ Failure Assessment
